# Genome–Wide Transcriptional Profiling and Functional Analysis Reveal That OsPHT4;4 Is Critical for the Growth and Development of Rice

**DOI:** 10.3390/ijms252313087

**Published:** 2024-12-05

**Authors:** Siyuan Li, Ruiyao Xu, Yaru Qiao, Yanglin Zhong, Xu He, Zhe Zhang, Shiqi Tian, Xue Yang, Lei Wu, Tiancheng Lu

**Affiliations:** 1 College of Life Sciences, Jilin Agricultural University, Changchun 130118, China; siyuanl@jlau.edu.cn (S.L.); 2211200113@mails.jlau.edu.cn (Y.Q.); 2211200128@mails.jlau.edu.cn (Y.Z.); wangshiwen@mails.jlau.edu.cn (X.H.); 20200957@mails.jlau.edu.cn (Z.Z.); 20220822@mails.jlau.edu.cn (S.T.); xyang@jlau.edu.cn (X.Y.); 2 Faculty of Agronomy, Jilin Agricultural University, Changchun 130118, China; xuruiyao@mails.jlau.edu.cn

**Keywords:** rice, *OsPHT4;4*, transcriptomic analysis, KEGG pathway, Pi metabolism

## Abstract

Phosphorus (P) is an essential macronutrient required for various vital processes in crop growth and development, including signal transduction, CO_2_ fixation, and photosynthetic phosphorylation. Phosphate transporters (PHTs) in plants play critical roles in the uptake, distribution, and internal transport of Phosphate (Pi). Among these transporters, the PHT4 family is widely distributed across plant species; however, the specific functions of many members within this family remain to be fully elucidated. This study focuses on unraveling the function of OsPHT4;4 in Pi utilization and photoprotection. The findings demonstrate that OsPHT4;4 acts as a low-affinity Pi transporter localized to the chloroplast membrane and reveal predominant expression of *OsPHT4;4* in leaves, with peak expression during tillering and clear induction by light, exhibiting circadian rhythmicity. The *ospht4;4* mutants display stunted growth. Transcriptomic analysis comparing *ospht4;4* mutants and wild-types (WT) identified 1482 differentially expressed genes (DEGs), including 729 upregulated genes and 753 downregulated genes. Kyoto Encyclopedia of Genes and Genomes (KEGG) pathway analysis reveals enrichment DEGs related to photosynthesis–antenna proteins, carbohydrate metabolism, and phenylpropanoid biosynthesis. These findings suggest that OsPHT4;4 plays crucial roles not only in photosynthesis but also in plant defense as an integral component involved in Pi metabolism.

## 1. Introduction

Phosphorus (P) is an essential element for various primary and secondary phosphorylation metabolites in plant cells and is also a component of a variety of nucleic acids and phospholipids [1]. The availability of phosphate(Pi) significantly influences plant growth and development, photosynthesis, and responses to various biotic and abiotic stresses [2]. Pi uptake by plants initially occurs through the root rhizosphere, followed by distribution of Pi within plant tissues, which is regulated by the plasmic membrane positioning phosphate transporter (PHTs) [3]. PHOSPHATE 1 (PHO1) specifically mediates the transport of Pi from the root system to the shoot [4]. Based on phylogenetic relationships, the PHT gene family can be classified into five distinct groups (PHT1–PHT5). Among them, PHT1 is localized to the plasma membrane and encodes the Pi transporter, primarily responsible for translocating Pi from the root cell’s plasma membrane to its interior. PHT2 is present in chloroplasts, PHT3 in mitochondria, and PHT5 in vacuoles; they facilitate Pi transport between the cytoplasm and various subcellular compartments. In contrast to other members, PHT4 localizes to either chloroplasts or Golgi apparatus. The PHT4 family exhibits a wide distribution across various plant species, including *Oryza sativa* (rice), *Arabidopsis thaliana*, *Populus simonii*, and *Physcomitrella patens* subsp. *californica* [5].

OsPHT4s have been demonstrated to enhance Pi transport across chloroplast or Golgi membranes in rice and may contribute to abiotic stress responses. PHT4 can be classified into six distinct groups (OsPHT4;1 to OsPHT4;6). In rice, OsPHT4;4 is localized on the chloroplast envelope [6]. This localization implies the involvement of OsPHT4;4 in chlorophyll function associated with Pi [5]. Furthermore, studies have revealed that the promoter region of *OsPHT4;4* contains a cis-element called the phosphoric acid hunger Response 1 (PHR1) binding sequence (P1BS: GnATATnC), which serves as a crucial regulator of Pi signaling and distribution and aids plants in tolerating Pi deficiency stress [7]. AtPHT4 has been investigated in *Arabidopsis thaliana* under iron-deficient conditions, where it was found that AtPHT4;4 interacts with bZIP58 to facilitate Pi transport and transport of chloroplast ascorbic acid, thereby mitigating damage caused by iron deficiency to photosynthesis processes [8]. The expression of *PHT4;4* varies in different tissues. In *Arabidopsis*, *AtPHT4;4* is mainly expressed in leaves and flowers, with minimal expression in the roots [9]. In Apple, *MdPHT4;4* is exclusively expressed in leaves [10].

Rice is one of the important food crops. Pi plays a critical role in regulating the accumulation of photosynthetic products by participating in both the light and dark reactions of photosynthesis. According to Li et al. [6], the OsPHT4 subfamily is involved in the translocation of Pi between cytoplasm and chloroplasts or Golgi apparatus, as well as in plant stress response. Despite their potential importance, the biological functions of OsPHT4 subfamily members remain underexplored. This study focuses on the native functions of OsPHT4;4 in rice, investigating its subcellular localization, spatiotemporal expression across different tissues, and Pi transport capabilities. We generated *ospht4;4* mutants in rice. The mutation was found to significantly impact the growth and development of rice compared to the wild-type. The *ospht4;4* mutants exhibited notable alterations in phenylpropanoid biosynthesis, photosystem function, and metabolic pathways associated with oxidative stress response. This study provides essential insights for further elucidating the biological role of *OsPHT4;4*.

## 2. Result

### 2.1. Bioinformatics Analysis of OsPHT4;4

**Figure 1:**
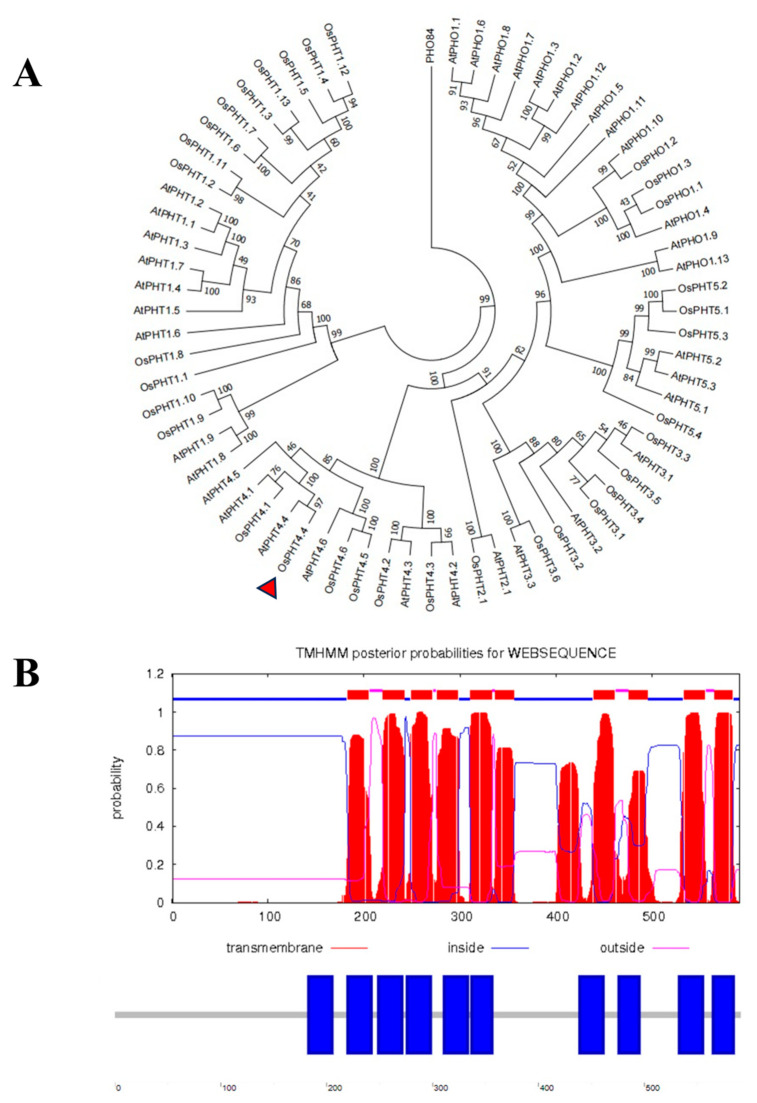
Bioinformatics analysis of OsPHT4;4 in rice and *Arabidopsis*. (**A**) Phylogenetic tree of phosphate transporters (PHT) family in rice and *Arabidopsis*. OsPHT4;4 is indicated by the red arrow. (**B**) Transmembrane prediction of OsPHT4;4. The JTT model implemented in the MEGA software was utilized to construct the phylogenetic tree, which underwent 1000 iterations.

Protein sequences of PHT from *Arabidopsis* and rice were aligned using ClustalW program, and the neighbor-joining method (NJ) was used in the construction of a phylogenetic tree using the MEGA7.0 program (1000 bootstrap replicates, neighbor–joining method) (Figure 1A). The phylogenetic analysis revealed that 33 phosphate transporter proteins in rice can be classified into six subfamilies: OsPHT1 subfamily (13 members), OsPHT2 subfamily (1 member), OsPHT3 subfamily (6 members), OsPHT4 subfamily (6 members), OsPHT5 subfamily (4 members), and OsPHO1 subfamily (3 members). Notably, the protein sequences of OsPHT4;4 and AtPHT4;4 clustered together with a high degree of homology, displaying a 97% similarity. This suggests a significant level of homology between these two genes. The transmembrane domains of the rice protein, OsPHT4;4, were predicted using TMHMM version 2.0 (http://www.cbs.dtu.dk/services/TMHMM/, accessed on 26 December 2023) and SMART (http://smart.embl.de/, accessed on 26 December 2023), as shown in Figure 1B. Ten transmembrane domains were identified at specific amino acid positions: 183–205, 220–242, 249–271, 276–298, 311–333, 337–356, 439–461, 476–495, 533–555, and 565–584.

### 2.2. The Temporal and Spatial Expression Pattern of OsPHT4;4 in Rice

**Figure 2:**
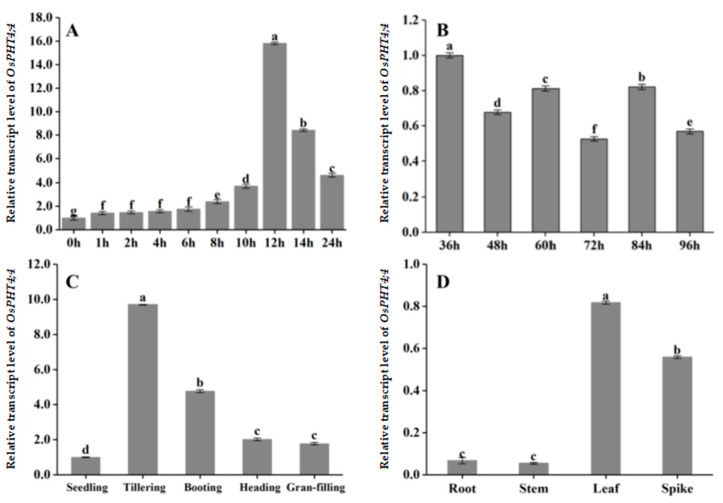
Spatiotemporal expression of rice *OsPHT4;4*. (**A**) Expression level of different times; (**B**) expression level of normal lighting; (**C**) expression level of different growth stages; (**D**) expression of different organizations. Data represent means ± SD (n = 3). The different letters above bars indicate statistically significant differences determined by one-way analysis of variance followed by Tukey’s multiple test (*p* < 0.05).

The temporal and spatial expression pattern of *OsPHT4;4* gene in rice was analyzed using quantitative polymerase chain reaction (qPCR) (Figure 2). The expression level of the *OsPHT4;4* gene exhibited a gradual increase upon exposure to light, reaching its peak at 12 h before subsequently declining at 14 and 24 h (Figure 2A). At its zenith, the expression level was approximately 15-fold higher than that observed at the onset of light exposure, indicating a significant disparity and highlighting substantial photosensitivity in gene regulation. Further investigation into the diurnal fluctuations over a span of 96 h was conducted to explore the gene’s expression dynamics (Figure 2B). Notably, under light conditions, significantly elevated levels of *OsPHT4;4* mRNA were detected at 36 h, 60 h, and 84 h compared to those observed at dark intervals such as 48 h, 72 h, and 96 h. This consistent pattern confirms that the transcriptional activity of *OsPHT4;4* follows a circadian rhythm. Additionally, the expression changes between 36 and 60 h and 48 and 72 h were significantly higher than those between 60 and 84 h and 72 and 96 h, indicating that the circadian expression of *OsPHT4;4* tends to stabilize under normal light conditions. To investigate the expression pattern of *OsPHT4;4* across different growth stages of rice, this study measured the expression level in five developmental stages: seedling, tillering, booting, heading stage, and grain filling(Figure 2C). The analysis revealed that the expression level of *OsPHT4;4* was highest at the tillering stage. The expression level in the grain filling stage was not significantly different from that in the heading stage. Overall, the *OsPHT4;4* exhibited a markedly higher expression during the tillering stage compared to other stages, highlighting its significant role in this particular phase of rice development.

Furthermore, the expression of *OsPHT4;4* in various tissues, including roots, stems, leaves, and spikes of rice plants during the ear extraction period, was analyzed (Figure 2D). The results demonstrate that this gene is expressed across all four tissues, albeit with varying levels. Notably, the highest expression of *OsPHT4;4* was observed in leaves, followed by spikes, while relatively lower expression levels were detected in roots and stems. These findings emphasize the significant role played by *OsPHT4;4* in contributing to and sustaining plant nutrient growth, particularly highlighting its importance in photosynthetically active and reproductive tissues.

### 2.3. Subcellular Localization of OsPHT4;4 Protein

**Figure 3:**
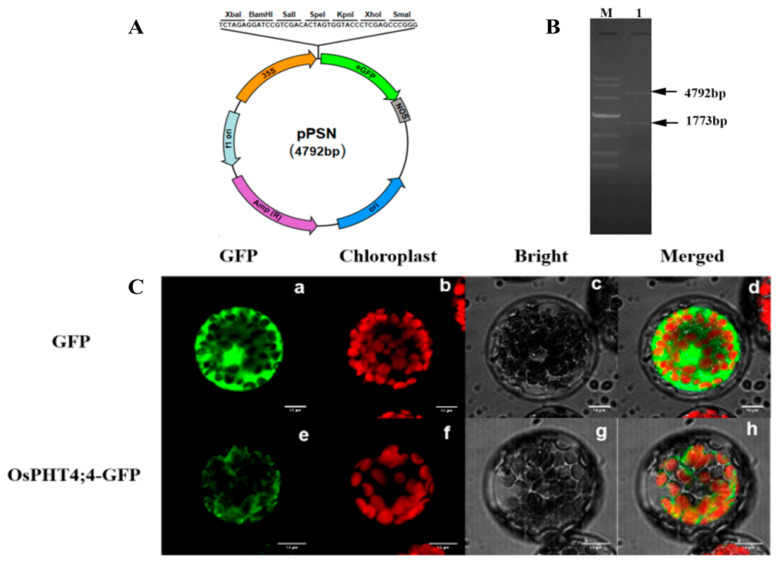
Construction of subcellular localization vector pPSN–*OsPHT4;4* and subcellular localization of *OsPHT4;4*–GFP. (**A**) pPSN vector map; (**B**) double restriction digestion verification of recombinant vector; (**C**) subcellular localization of OsPHT4;4–GFP. (**a**–**d**) represent control GFP protein localization; (**e**–**h**) represent GFP with OsPHT4;4 fusion protein localization. (**a**,**e**) represent GFP green fluorescence; (**b**,**f**) represent Chloroplast autofluorescence; (**c**,**g**) represent bright field observation; (**d**,**h**) represent superimposed picture of the three. The green signal indicates green fluorescent protein (GFP) and the red signal indicates chloroplast autofluorescence. Scale bars:10 μm.

To investigate the subcellular localization of OsPHT4;4, we amplified its full-length gene using primers based on the nucleic acid sequence (LOC_Os09g39680) and cloned its coding sequence (CDS), excluding the stop codon, into pPSN–GFP fusion expression vector via XbaI and XhoI restriction enzymes (Figure 3A). The successful construction of pPSN–*OsPHT4;4* subcellular localization vector was confirmed by double enzyme digestion, which revealed two distinct bands: a 1773 bp band corresponding to the target gene and a 4792 bp band corresponding to the vector (Figure 3B).

Subsequently, *Arabidopsis* protoplasts were transfected with pPSN–*OsPHT4;4* or control vector pPSN using PEG–Ca^2+^ mediated transformation for subcellular localization studies. Laser confocal microscopy showed that GFP fluorescence from OsPHT4;4 coincided with chloroplast autofluorescence, indicating its localization on the chloroplast membrane (Figure 3C).

### 2.4. Functional Complementation of OsPHT4;4 Gene in Yeast Mutant

**Figure 4:**
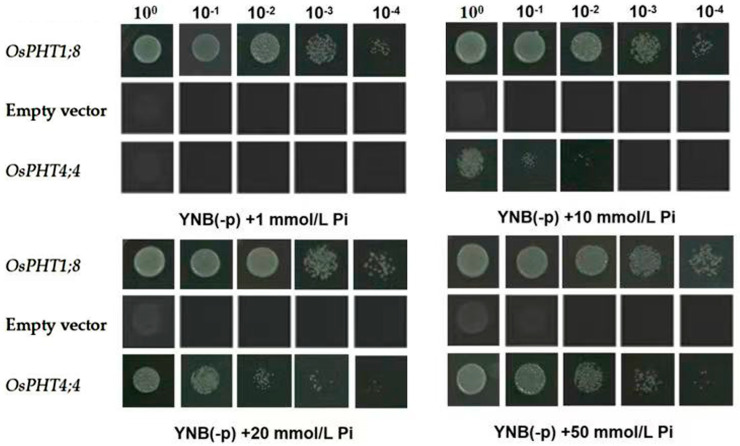
Complementary test of *OsPHT4;4* yeast mutant PAM2. The empty carrier transformation strain is a negative control (empty vector), and the pYES2–*OsPHT1;8* carrier transformation strain is a positive control (*OsPHT1;8*). PYES2–*OsPHT4;4* transformed yeast cells (*OsPHT4;4*) are cultured to OD600 = 1 in a synthetic dropout (Trp/Ura) culture medium containing Gal. Apply 5 μL culture solution or 10 times gradient dilution to a flat plate containing 1 mM, 10 mM, 20 mm, and 50 mM Pi for plate coating results.

To assess the Pi transport capabilities of the OsPHT4;4 protein, we employed a complementation assay using the yeast mutant PAM2, deficient in high-affinity Pi transporters. This particular yeast strain lacks PHO84 and PHO89, which are responsible for high-affinity Pi translocation, as well as the ability to synthesize Ade2 and Ura3 amino acids. Consequently, its growth is hindered in low-Pi media without supplementation of Ade2 and Ura3. The expression vector pYES2, containing a GAL1 promoter upstream of the multiple cloning site, enables inducible expression of exogenous genes through galactose induction and harbors essential genes for Ura3 amino acid synthesis. As a positive control, OsPHT1;8, known for its high-affinity Pi transport, was utilized, while an empty pYES2 vector served as a negative control. The three yeast strains harboring the respective recombinant plasmids were serially diluted and cultured on low-Pi deficient media, as illustrated in Figure 4. The strain containing OsPHT1;8 exhibited robust growth under varying Pi concentrations, demonstrating its strong viability. In contrast, the strain with the empty vector showed no growth at Pi concentrations of 1 mM, 10 mM, and 20 mM, displaying only marginal growth at the baseline concentration of 50 mM Pi. Interestingly, the strain expressing the OsPHT4;4 protein demonstrated slightly slower growth compared to the positive control but displayed significantly enhanced growth compared to the negative control when starting from a Pi concentration of 10 mM and increasing thereafter. These results obtained from the yeast complementation assay suggest that OsPHT4;4 possesses functional Pi transport capability with a lower affinity than OsPHT1;8.

### 2.5. Phenotype of Rice Plants Under Pi Concentration Gradient

Using CRISPR/Cas9 genome editing technology, we successfully constructed the knockout vector pCBSG032–*OsPHT4;4*. The *OsPHT4;4* transgenic plants were transformed via Agrobacterium-mediated transformation. Genomic DNA was extracted from the T0 generation of transgenic plants, and, following target-specific and positive PCR amplification and sequencing, *ospht4;4* mutants were generated. Sequence analysis revealed an insertion of a single base at the SG position in the gene, resulting in premature termination of the encoded protein. The construction and verification of *ospht4;4* mutants are illustrated in Figure S1.

**Figure 5:**
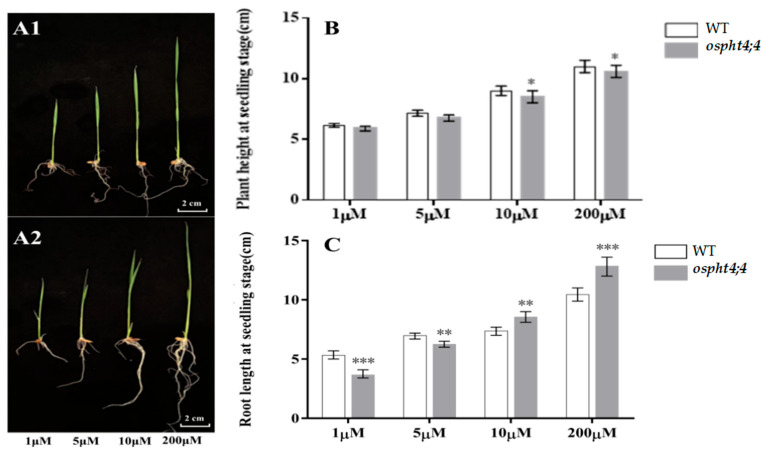
Single plant phenotype of wild-type rice and *ospht4;4* mutants under Pi concentration gradient. (**A1**) Single wild-type plant; (**A2**) single *ospht4;4* mutant plant; (**B**) plant height of wild-type and *ospht4;4* mutants under Pi concentration gradient; (**C**) plant root length of wild-type and *ospht4;4* mutants under Pi concentration gradient. The levels of statistical significance are denoted by different asterisks: * *p* < 0.05, ** *p* <0.05, *** *p* <0.01, (Student’s *t*-test), n = 5. WT refers to wild-type rice, while *ospht4;4* represents the *ospht4;4* mutants.

We subsequently examined the seedling phenotype of both *ospht4;4* mutants and wild-type plants (WT). Observations of individual plant phenotypes (Figure 5A1,A2) under Pi concentration gradients of 1 μM, 5 μM, 10 μM, and 200 μM indicated no significant difference in plant height between *ospht4;4* mutants and wild-type plants. However, the root length of *ospht4;4* mutants was notably greater than that of the wild-type plants at Pi concentrations of 10 μM and 200 μM. Contrary to this, data from Figure 5B show that the height of wild-type plants exceeds that of the *ospht4;4* mutants across all tested Pi concentrations, with significant differences observed at 10 μM and 200 μM. The pattern of root length changes depicted in Figure 5C differs markedly from plant height trends. At lower Pi concentrations (1 μM and 5 μM), wild-type plants exhibited significantly longer roots compared to *ospht4;4* mutants. Conversely, at higher concentrations (10 μM and 200 μM), the root lengths of *ospht4;4* mutants were substantially greater than those of wild-type plants. These findings preliminarily indicate that OsPHT4;4 plays a role in Pi transport in rice plants, particularly under high Pi conditions (>10 μM). The impact on Pi dynamics in *ospht4;4* mutants became more pronounced. The reduced Pi transport capacity in *ospht4;4* mutants necessitates enhanced root development to acquire sufficient Pi for normal plant growth.

### 2.6. Transcriptomics Analysis of ospht4;4 Mutants and wild-types

**Figure 6:**
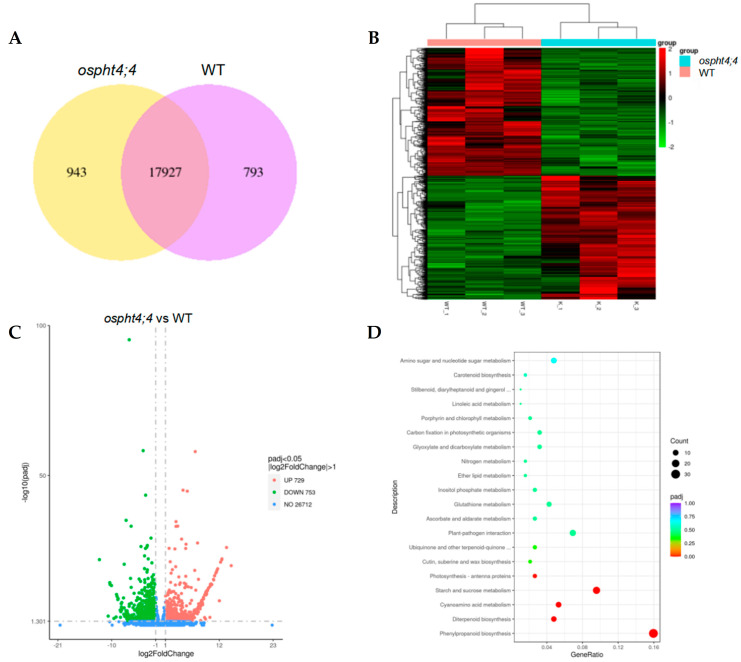
Transcriptomic analysis of *ospht4;4* mutants and wild-type rice. (**A**) Venn diagram of DEGs; (**B**) volcanic map of DEGs; (**C**) clustering heat map of DEGs; (**D**) KEGG enrichment analysis was performed on the differentially expressed genes (DEGs), with the color of each dot indicating the adjusted *p*-value (padj). The intensity of the red color reflects the significance level of enrichment, while the size of each dot represents the number of enriched DEGs. In this study, WT refers to wild-type rice, and *ospht4;4* denotes *ospht4;4* mutants.

The Illumina NovaSeq 6000 platform was employed for transcriptome sequencing of wild-type rice and *ospht4;4* mutants. As depicted in Figure 6A, the Venn diagram reveals a total of 17,927 genes that are commonly expressed in both the *ospht4;4* mutants and wild-type rice samples, with 793 genes uniquely expressed in the wild-type and 943 genes exclusively expressed in the *ospht4;4* mutants. The distribution of differentially expressed genes (DEGs) is illustrated in Figure 6B using a volcano plot, highlighting significant disparities in gene expression between the *ospht4;4* mutants and wild-types. A comprehensive analysis identified a total of 1482 DEGs, among which 729 exhibited increased expression, while 753 showed decreased expression levels compared to the wild-types. The clustering heat maps presented in Figure 6C demonstrate substantial variations in gene expression patterns between the two varieties. To elucidate their functional roles within various signaling pathways, KEGG pathway enrichment analysis was conducted on these DEGs, resulting in identification of 82 significantly enriched signaling pathways (Figure 6D). Notably, five pathways, including phenylpropanoid biosynthesis, diterpenoid biosynthesis, cyanogenic amino acid metabolism, starch and sucrose metabolism, and photosynthetic antenna proteins, were found to be significantly enriched (Padj < 0.05). Detailed statistics regarding key enriched genes within each KEGG pathway can be found in Table S2.

## 3. Discussion

Pi is a critical component of chloroplasts, directly influencing the structure and function of photosynthetic organs [11]. A deficiency in Pi markedly impairs plant photosynthesis, as evidenced by significantly reduced leaf net photosynthesis rates in species such as *Arabidopsis* [12], soybeans [13], and rice [14]. Among the Pi transporters, some members of the PHT4 family are localized to the chloroplast [15] and are hypothesized to play a pivotal role in Pi translocation within these organelles. In this study, subcellular localization tests and bioinformatics analysis confirmed that OsPHT4;4 is a chloroplast membrane protein with 10 transmembrane regions. Phosphate transport proteins are categorized based on their Pi affinity into high and low affinity types [16]. Yeast functional complementarity assays demonstrated that yeast strains expressing *OsPHT4;4* can survive in Pi -deficient media, albeit with slower growth compared to strains expressing the high-affinity transporter OsPHT1;8. This finding suggests that OsPHT4;4 functions as a low-affinity Pi transporter in the chloroplast membrane. Furthermore, CRISPR/Cas9 gene editing was employed to generate a rice mutant lacking the *OsPHT4;4* gene. The *ospht4;4* mutants exhibited stunted growth compared to the wild-types, preliminarily indicating that the disruption of *OsPHT4;4* affects plant growth and development to a significant extent.

Phylogenetic analysis demonstrated that the OsPHT4;4 protein sequence shares 97% similarity with *Arabidopsis* AtPHT4;4, suggesting a close evolutionary relationship and potentially similar biological functions. Previous research by Guo et al. [7] indicated that in *Arabidopsis*, *AtPHT4;1* and *AtPHT4;4* are light-induced and predominantly expressed in photosynthetic tissues, with *AtPHT4;1* and *AtPHT4;2* showing circadian rhythm fluctuations. Our qPCR results confirm that *OsPHT4;4* expression in rice is also light–induced and exhibits circadian rhythm changes. Li et al. [6] reported that transcription levels of the *OsPHT4;4* gene vary across different rice organs. During the nutritional stage, expression levels of *OsPHT4;1*, *OsPHT4;3*, *OsPHT4;4*, and *OsPHT4;5* are notably weak in the roots, with higher levels observed in the stems. *OsPHT4;2*, *OsPHT4;6–1*, and *OsPHT4;6–2* exhibit high expression in the stems. In contrast, during the flowering and grain–filling stages, all *OsPHT4* genes show elevated transcription levels in the leaves, whereas in the roots, except for *OsPHT4;2* and *OsPHT4;6*, the levels remain very low. At the spikelet filling stage, transcription levels of *OsPHT4;2* and *OsPHT4;6–1* surpass those of *OsPHT4;1*, *OsPHT4;3*, and *OsPHT4;4*. Additionally, expression of *OsPHT4;1*, *OsPHT4;3*, and *OsPHT4;4* is higher in new leaves compared to old leaves. Our findings highlight that *OsPHT4;4* is primarily expressed in the leaves, with gene expression showing an initial increase and subsequent decline throughout the reproductive period. The peak expression occurs during the tillering stage, aligning with predictions from the RiceXPro and BAR databases (Figure S2).

Transcriptional analysis of wild-type rice and *ospht4;4* mutants in this study identified a total of 1482 differentially expressed genes (DEGs). KEGG pathway analysis revealed that these DEGs were predominantly enriched in the pathways related to phenylpropanoid biosynthesis, diterpenoid biosynthesis, photosystems, antioxidative stress, and anti-pathogens. All the differences are related to the change in Pi transport of the chloroplast envelope membrane.

Phenylalanine is used as the initial raw material to enter the phenylpropane metabolic pathway and is further transformed into coumarin and chlorogenic acid through intermediate products such as trans-cinnamic acid, coumaric acid, ferulic acid, and erucinic acid [17]. Dong et al. [18] reviewed the phenylpropanoid metabolism to plant development and plant-environment interactions. The lignin pathway is a branch pathway of phenylpropane metabolism. Lignin mainly accumulates in plant secondary cell walls, providing mechanical support for plants and participating in the formation of conduits to transport water and mineral elements. In addition, lignin is also involved in another development, resistance to pathogen invasion, resistance to herbivore feeding, and resistance to abiotic stress. In the present study, the biosynthesis of lignin was reduced in *ospht4;4* mutants, suggesting the disease resistance went down. Flavonoids act as antioxidants to remove reactive oxygen species from plants. Flavonoids, flavonols, and anthocyanins are involved in plant defense against pathogens and herbivores. In the present study, the biosynthesis of flavonoid compounds was downregulated. In addition, a coumarin biosynthesis bypass went up in *ospht4;4* mutants, suggesting a complicated biochemical reaction. The underlying mechanism leading to such results is complicated in *ospht4;4* mutants. In order to adapt to the changing environment, plants need to constantly adjust the distribution of energy and metabolites in different development processes to maintain vigorous growth and survival under adverse conditions. Metabolic flow reorientation can occur between primary metabolism and secondary metabolism, between phenylpropane metabolism and other secondary metabolic pathways, between different branches of the phenylpropane metabolism pathway, and between different secondary branches of the same branch, so as to maintain the dynamic balance of phenylpropane metabolism, maintain the vigorous growth of plants, and resist biological and abiotic stress. In the present study, a certain fact was that the changes in phenylpropanoid metabolism in *ospht4;4* mutants were closely related to the *PHT4;4* gene knockout event, resulting in metabolic flow reorientation, and the metabolic flow reorientation was related to the change in Pi transport of the chloroplast envelope membrane.

GA can promote plant growth and flowering. In the present study, the diterpenoid biosynthesis of GA was upregulated, whereas the *ospht4;4* mutants displayed the phenotypes of slow growth and flowering. The functions of GA were hampered. At the same time, another flowering regulation protein ELF4 (LOC107278437) was also putative to be upregulated. This study has suggested that two ELF protein family members, EFL1 and EFL3, are involved in flowering time regulation in *Arabidopsis* [19]. This implies the EFL families may be far involved in flowering time regulation, and it was putative that GA may promote ELF4 expression. On the other hand, the protein far1-related sequence 5 (LOC107281147) and ripening-related protein 3-like (LOC4348971) were putative to be downregulated. The two genes are involved in plant growth, development, and fruit time [20]. Their downregulation should contribute to the *ospht4;4* mutant phenotypes of slow growth and flowering.

Five genes associated with the photosynthesis–antenna protein pathway (LOC4324705, LOC4324599, LOC4343583, LOC4336028, and LOC4347166) exhibited an upregulated expression (*p* > 0.05). Additionally, multiple genes involved in the photosynthetic carbon fixation pathway (dosa00710) were also upregulated. These findings indicate that the photosynthesis of *ospht4;4* mutants is significantly impacted, suggesting that the cells might accelerate the photosynthetic reaction process to compensate for the cellular damage caused by Pi deficiency. Photosynthesis involves a dark reaction where plants use ATP and NADPH to convert CO_2_ into organic substances such as glucose, with sucrose typically being the final product in most plants. The metabolism of sucrose not only generates hexoses, which are crucial for the synthesis of cellulose, starch, fructans, proteins, and antioxidants, but also produces signaling molecules like sucrose itself, glucose, fructose, and trehalose-6-phosphate (T6P). These sugar signals, or the signaling effects of the metabolic process itself, are intricately linked with other signaling pathways, including those mediated by hormones and redox processes [21].

The response to oxidative stress (GO0006979) was a dominant GO term, and it was putative to go down. A series of peroxidases was involved in going down. The biosynthesis of flavonoids (LOC4336764 flavanone 3-dioxygenase 2) was also putatively reduced. These findings suggest that the lack of *PHT4;4* affected the response to oxidative stress in rice leaves, and the reduced response to oxidative stress was related to the change in Pi transport of the chloroplast envelope membrane.

Upon the plant–pathogen interaction and disease resistance, many genes that are enriched in disease resistance and response to various stresses were downregulated in *ospht4;4* mutants, and two genes of protein far1-related sequence 5 (LOC107281147) for plant growth and development and zinc finger protein 385A (LOC4348948) for transcription factor were also downregulated in *ospht4;4* mutants. At the same time, in the diterpenoid biosynthesis pathway, the enzymes for biosynthesis of anti-pathogenic oryzalexin D and oryzalexin E went down [22]. The abilities of disease resistance and adaptation to various stresses are critical for plants [23,24,25]. *ospht4;4* mutants apparently affected the disease resistance of the rice plant, indicating the disorder in Pi transport of the chloroplast envelope membrane led to a severely adverse event. Interestingly, we found the anti-pathogenic WRKY12/PR1/oryzalexin pathway was putative to be downregulated in *ospht4;4* mutants. Zhang et al. [26] reported the OsWRKY family members of OsWRKY21 and OsWRKY108 functioned redundantly to promote Pi accumulation through maintaining the constitutive expression of *OsPHT1;1* under phosphate–replete conditions. This suggests the OsWRKY families are deeply involved in Pi transport and accumulation in rice tissues. In the present study, we suggest the WRKY24/PR1/oryzalexin pathway may be the underlying molecular mechanism by which wild-type rice leaves resist pathogens. This is a putative finding of the present study worthy of verification. On the other hand, the CaM/CML-reinforced cell walls were an unaffected anti-pathogenic mechanism in *ospht4;4* mutants.

## 4. Materials and Methods

### 4.1. Plant Materials and Growth Conditions

The rice variety used in this study is *Oryza sativa* L. ssp. *japonica* cv Nipponbare (BIOGLE GeneTech, Nanjing, China). The plants were cultivated under controlled conditions with a constant temperature of 25 °C, relative humidity of 60%, and a photoperiod of 14 h light followed by 10 h darkness. Each pot initially contained five rice seeds, from which three seedlings were ultimately selected for further growth. Following the development to the two-leaf–one-heart stage in a hydroponic setup, seedlings were transferred to Hogland nutrient solution with varied Pi concentrations. The culture solution was changed every three days. After 21 days of growth, plants were kept in darkness for 3 days before reintroduction to light. Leaf samples were collected at intervals of 0 h, 2 h, 4 h, 6 h, 8 h, 10 h, 12 h, 14 h, and 24 h, respectively, for diurnal rhythm expression. The expression levels of the *OsPHT4;4* gene were analyzed at five developmental stages: seedling, tillering, booting, heading, and grain filling. Additionally, *OsPHT4;4* expression was assessed in different tissues and organs, including roots, stems, leaves, and spikes, during the heading period.

The experimental setup involved potted plants grown in a drought-resistant greenhouse at the National Crop Variety Approval Characteristic Appraisal Station of Jilin Agricultural University. Each pot was filled with 11 kg of soil, with 3 evenly distributed holes and 5 grains per hole, with only one seedling being retained per hole. The nutrient management was applied across all treatments, with each pot receiving a single application of 5.65 g of compound fertilizer (N–P–K:20–12–14) as a base fertilizer at one time. Watering was applied once before sowing, and a thin layer of soil (1–2 cm) was applied after sowing. All subsequent management practices were consistent across all treatments.

### 4.2. Bioinformatics Analysis of Rice Pi Transporters

The protein sequences of each member of the PHT gene family were retrieved from the *Arabidopsis* TAIR database (https://www.arabidopsis.org/, accessed on 26 December 2023) and utilized as templates for homologous comparison against the rice genome database on the Phytozome website (https://doe.gov/, accessed on 26 December 2023). This analysis successfully identified all members of the PHT gene family. Subsequently, the ClustalW program in BioEdit software (v7.0.5) was employed to align the PHT protein sequences from rice and *Arabidopsis*. A phylogenetic tree was constructed using MEGA software (v7.0.26) with the Jones–Taylor–Thornton (JTT) model employing 1000 bootstrap replications for evolutionary analysis. Transmembrane regions of the OsPHT4;4 protein in rice were predicted using online tools TMHMM and SMART. Expression patterns of *OsPHT4;4* at different growth stages and in various tissues were analyzed by querying accession number LOC_os09g39680 in RiceXPro database (https://ricexpro.dna.affrc.go.jp/quick–guide.html, accessed on 11 January 2024) and BAR database (http://bar.utoronto.ca/, accessed on 25 January 2024).

### 4.3. RNA Isolation and Quantitative Real-Time PCR (qRT–PCR)

The TAKARA Trizol Kit (TAKARA, Beijing, China) was used to extract total RNA from plant samples, following the manufacturer’s instructions. To synthesize the first-strand cDNA, 2 µg of total RNA was treated with DNA enzyme using the PrimeScriptTMRT reagent Kit with gDNA Eraser (Takara, Japan). The reaction was carried out in a total volume of 20 µL, and the synthesized cDNA was stored at −20 °C for further usage.

qRT–PCR analysis was performed on an ABI Step One Plus system (Roche, Basel, Switzerland) using 384-well plates. Each 25 µL reaction mixture included 1 µL cDNA and 2 µL of each gene-specific primer (OsPHT4;4QF and OsPHT4;4QR from primers in Table S1), along with 10 µL master mix (SYBR Green PCR Mastermix, Takara, Japan). The rice EF1α gene (OsEF1αF and OsEF1αR from Primers in Table S1) served as a reference gene. Gene expression levels were calculated using the 2^−ΔΔCt^ method [27]. Each qRT–PCR experiment consisted of three biological replicates and three technical replicates. Statistix (v 8.1) software was used to analyze the significance of the data.

### 4.4. Subcellular Localization of Genes

For subcellular localization analysis, the open reading frame (ORF) of *OsPHT4;4* (primers OsPHT4;4F and OsPHT4;4R from Table S1) was amplified and cloned into a pPSN vector containing 35S promoter and green fluorescent protein (GFP) gene fragments. The GFP was fused to the C-terminals of these genes. The resulting GFP fusion plasmids were introduced into protoplasts using polyethylene glycol (PEG)-mediated transformation. Protoplast preparation and transfection followed established protocols [28,29]. Microscopy images were acquired using a laser scanning confocal microscope (Leica TCS SP5, Leica, Wetzlar, Germany). Excitation and emission wavelengths Ex488/Em500–550, Ex516/Em600–650, and Ex587/Em602–652 were employed for visualizing GFP fluorescence, autofluorescence of chlorophyll, and red fluorescent protein (RFP), respectively.

### 4.5. OsPHT4;4 Yeast Function Complementarity Test

The primers OsPHT1;8F and OsPHT1;8R (see Schedule 1) were used to amplify the target gene *OsPHT1;8* from rice RNA as a template, which was subsequently connected, transformed, identified, and sequenced for control purposes. The pYES2 plasmid was enzymatically cut using SacI and XhoI for *OsPHT4;4* insertion and HindIII and XbaI for *OsPHT1;8* insertion, respectively. The resulting target fragments of *OsPHT4;4* and *OsPHT1;8* were then inserted into the yeast expression vector pYES2 to construct the yeast expression vectors pYES2–*OsPHT4;4* and pYES2–*OsPHT1;8* accordingly. These constructed vectors, along with empty-loaded pYES2, were transformed into the yeast mutant strain PAM2 receptor cells for further experiments. Afterwards, positive transformation strains were selected by culturing them in liquid medium until OD600 ≈ 1 was reached. Subsequently, 1 mL of bacterial solution was collected, centrifuged at 6000 rcf for 5 min, washed with 1 mL ddH_2_O, and again followed by another centrifugation at 6000 rcf for 5 min. To evaluate their performance under Pi-deficient conditions, the diluted bacterial solutions (10-fold serial dilutions: 10^0^, 10^−1^, 10^−2^, 10^−3^, 10^−4^, 10^−5^) were applied onto Pi-deficient yeast culture media containing different concentrations of Pi (i.e., 1 mM, 10 mM, 20 mM, and 50 mM). Finally, the cultures were incubated at 30 °C and photographed.

### 4.6. Construction of ospht4;4 Mutant Strains

The gRNA target sequence was used to generate the Oligo sequence online through the website (https://www.biogle.cn/, accessed on 12 January 2023). The synthesized Oligo was dissolved in ddH_2_O to a concentration of 10 μM, and the wild *OsPHT4;4* sequence was amplified as a template for preparing the Oligo dimer. Subsequently, the constructed Oligo dimer was inserted into the pCBSG032 vector and transformed into DH5α recipient cells. The positive plasmid obtained from sequencing was then transformed into Agrobacterium EHA105. After transformation, rooting, and verification detection, positive transgenic plants were obtained and subsequently transplanted and propagated to obtain T0 seeds. In this study, PCR method and sequencing were employed for detecting positive transgenic events using specific primers, including OsgRNA–F, OsgRNA–R for gRNA amplification, KO–OsPHT4;4F, and KO–OsPHT4;4R for template amplification of the *ospht4;4* mutant region, as well as BAR–3F and BAR–3R3 primers (refer to Schedule 1) for further confirmation.

### 4.7. Phenotypic Observation of Rice Plants Under Different Pi Concentrations

Select wild-type seeds and T2 generation mutant strains of rice *OsPHT4;4* with complete phenotypes and consistent sizes and sterilize them with 6% NaClO for 1 h, followed by rinsing with deionized water three times for 5 min each time. After moistening the seeds with water, place them in a constant temperature incubator at 37 °C to accelerate germination. After 24 h, remove the seeds and evenly spread them on a large plate, soaking them in deionized water and covering them with filter paper. Keep the plate in darkness at a temperature of 24 °C in the tissue culture room for two days. Once germinated, sow the seedlings evenly on sterile filter paper placed inside a culture dish and cultivate them using deionized water in a tissue culture chamber. When both *ospht4;4* mutants and wild-type rice reach the two-leaf–one-heart stage, transfer them to barrels containing rice nutrient solution under four different Pi concentration gradients (1 μM, 5 μM, 10 μM, and 200 μM) for one week. Capture photographs and measure plant height as well as root length. Rapidly freeze leaves from both *ospht4;4* mutant and wild-type rice using liquid nitrogen before storing them at −80 °C.

### 4.8. Transcriptome Sequencing of ospht4;4 Mutants and wild-type Rice

The total RNA of rice seedling leaves was extracted using the TRIzol method. Subsequently, RNA integrity was assessed, and a cDNA library was constructed using the Agilent 2100 biological analyzer. Sequencing was performed on the Illumina NovaSeq 6000 platform (Novegene Technology Co., Ltd. of Beijing, China). Raw data were filtered, and Clean Reads were obtained for further analysis using Fastp tool (v.0.21.0). Comparison between Clean Reads and the reference genome was conducted using HISAT2 software, followed by assembly and quantification with StringTie software (v.2.2.0). Differential expression genes were identified using DESeq2 based on selection criteria of |log_2_(FoldChange)| ≥ 1 and padj ≤ 0.05. GO function enrichment analysis and KEGG pathway enrichment analysis of DEGs were performed using ClusterProfiler v4.0. 

### 4.9. Statistical Analysis

The data analysis was conducted using SPSS Statistical 20.0 software, and group differences were assessed using Duncan’s test. Statistically significant differences were denoted by an asterisk (*) for *p* ≤ 0.05. The significance of the variations among the experimental groups was calculated through one-way analysis of variance (ANOVA) followed by Tukey’s multiple test (*p* < 0.05). The letters above the columns indicate significant distinctions among the different options. The data are presented as means ± SD.

## 5. Conclusions

In conclusion, OsPHT4;4 is a low-affinity Pi transporter localized in the chloroplast membrane, which is mainly expressed in leaves and peaks in the tiller stage. In addition, its expression is regulated by light, showing a circadian rhythm. Compared with wild-type rice, *ospht4;4* mutant rice grows slowly and weakly. Transcriptome analysis of *OsPHT4;4* showed reduced response to oxidative stress and ability to resist and adapt to stress. The biosynthesis of lignin, flavonoids, and glutamic oxalosides were downregulated. Speculated in *OsPHT4;4* rice leaves, the anti–pathogenic WRKY24/PR1/oryzalexin pathway was reduced. Therefore, the WRKY24/PR1/oryzalexin pathway may be an *OsPHT4;4* important molecular mechanism of resistance of wild-type rice leaves to pathogenic bacteria. The biosynthesis of gibberellin is thought to be upregulated, and the function of gibberellin is impeded. It is hypothesized that the photosystem in rice leaves is upregulated to rebalance; in particular, the OsZIP family is thought to prevent the downregulation of photosynthesis or improve chloroplast development. This biological information is related to the changes of Pi transport in the chloroplast envelope. The results are helpful to understand the function of *OsPHT4;4* in rice and guide the next *OsPHT4;4* related research, which is conducive to rice breeding.

## Data Availability

Data will be made available upon request.

## Supplementary Materials

The following supporting information can be downloaded at: https://www.mdpi.com/article/10.3390/ijms252313087/s1.
