# Supplementary material for: Genome–Wide Transcriptional Profiling and Functional Analysis Reveal That OsPHT4;4 Is Critical for the Growth and Development of Rice
Source: Int J Mol Sci. 2024 Dec 5;25(23):13087. doi: 10.3390/ijms252313087 (PMC11642169; doi:10.3390/ijms252313087)
Supplement: Supplementary file 1 [file ijms-25-13087-s001.zip › ijms-3319085-supplementary.docx]

**
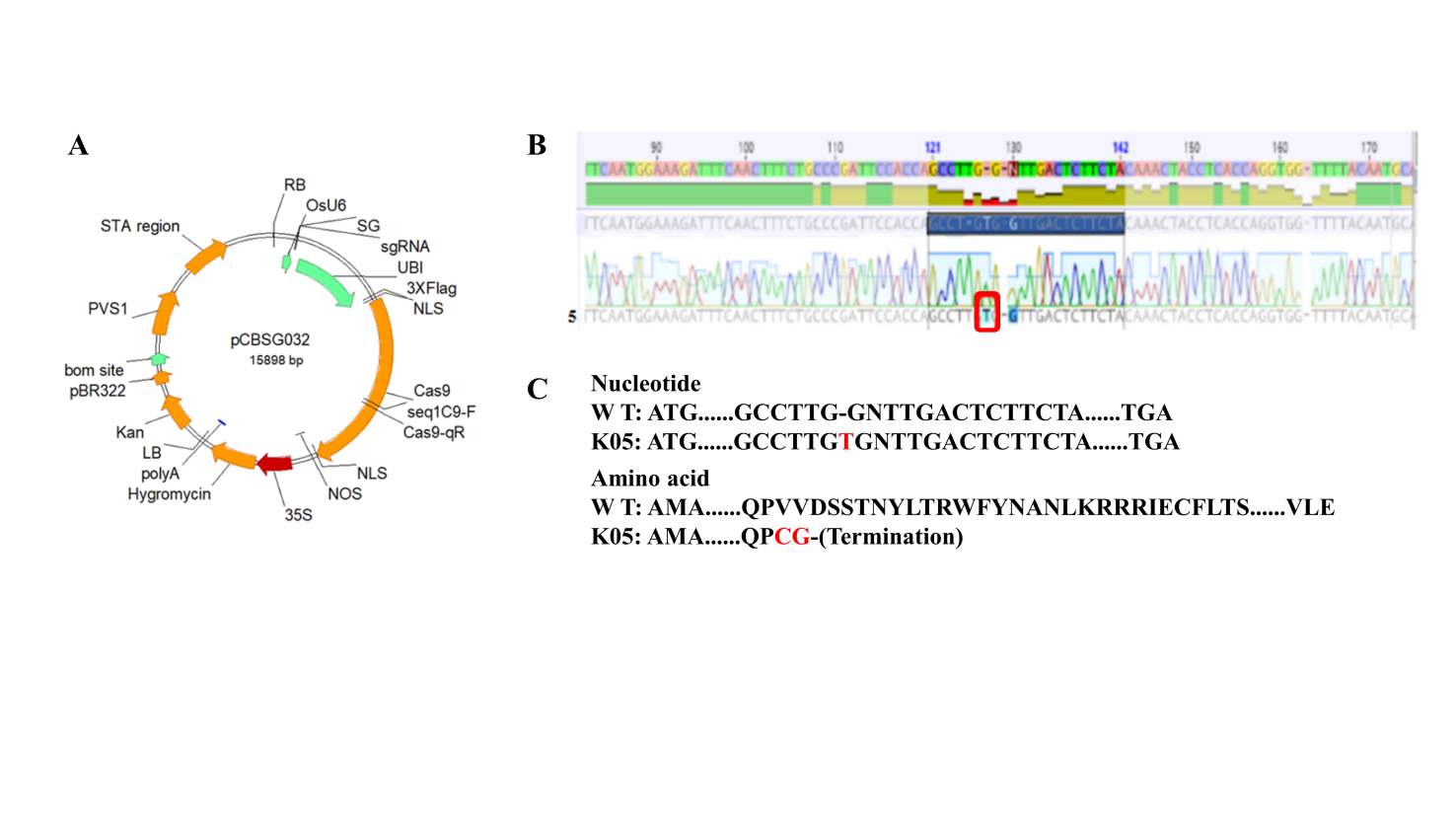
Figure S1.** Construction of gene knockout vector for *OsPHT4;4* and acquisition of transgenic plants. (A) pCBSG032 vector map; (B) Sequencing result analysis; (C) nucleotide and amino acid sequence comparison.


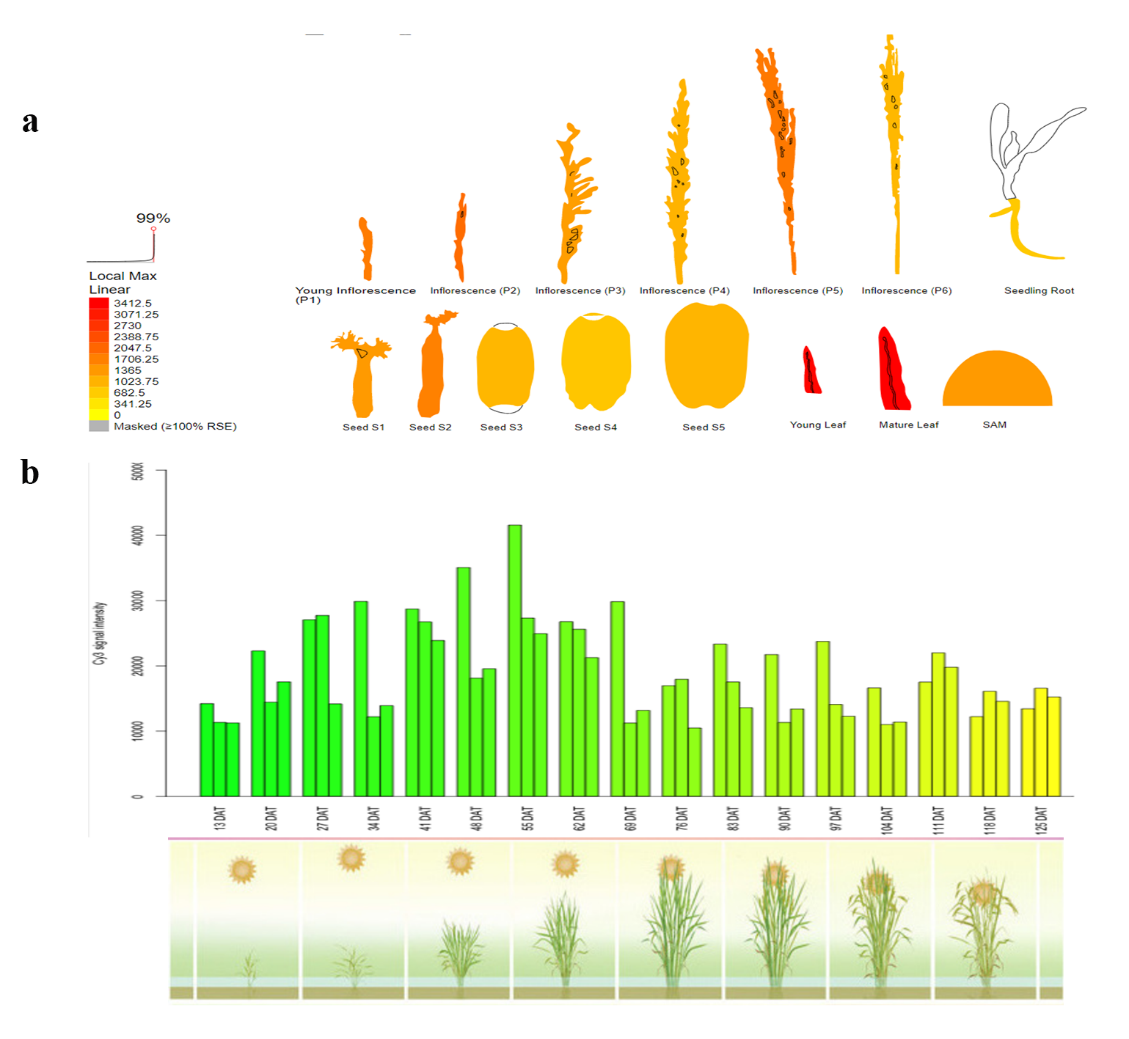


**Figure S2.** Prediction of *OsPHT4;4* gene expression in different tissues and organs and throughout the growth period. (A) BAR database expression prediction in different tissues; (B) RiceXPro database expression prediction in the whole growth period.

Table S1: Primers

| Primer name | sequence |
| --- | --- |
| OsPHT4;4F | CAGCAGTTCCCTAAGCCGAT |
| OsPHT4;4R | AAGAAGGAGGACTGGATTAGGC |
| OsPHT1;8F | ATGGCGCGGCAGGAGCAGC |
| OsPHT1;8R | CTACGCCGTCTGCGGCCG |
| Y-OsPHT4;4F | TGCTCTAGAATGGCTATGGGGGCCGTG |
| Y-OsPHT4;4R | CCGCTCGAGTTCAAGAACTTTCTCTCCAGTTGAGAAG |
| JM-OsPHT4;4F | CCCAAGCTTATGGCTATGGGGGCCGTGC |
| JM-OsPHT4;4R | TGCTCTAGATCATTCAAGAACTTTCTCTCC |
| JM-OsPHT1;8F | CGAGCTCATGGCGCGGCAGGAGCAGC |
| JM-OsPHT1;8R | CCGCTCGAGCTACGCCGTCTGCGGCCG |
| OsEF1αF | ACATTGCCGTCAAGTTTGCTG |
| OsEF1αR | AACAGCCACCGTTTGCCTC |
| OsPHT4;4QF | GCGATGGACAGTCGTTCT |
| OsPHT4;4QR | AAGGAGGACTGGATTAGGC |
| OE35S+OsPHT4;4F | CGAGCTCCATGGAGTCAAAGATTCAAATAGAG |
| OE35S+OsPHT4;4R | CCCAAGCTTTCATTCAAGAACTTTCTCTCC |
| OsgRNA-F | CCTGGCTGTATTTGGTGGTTG |
| OsgRNA-R | ACGACCGGGTCACGCTGCACCT |
| UP | TGTGTGAGAAGAGTCAACCACAGGC |
| LOW | AAACGCCTGTGGTTGACTCTTCTCA |
| KO-OsPHT4;4F | CTCACCTCCCACCCGAGATA |
| KO-OsPHT4;4R | TAGTCTGGCTGGACACAAGC |
| BAR-3F | CCATCGTCAACCACTACATCGAGACA |
| BAR-3R | GTGTAGAGCGTGGAGCCCAGTCCC |

Table S2: Statistics of Main Enriched Genes in KEGG Pathways

| KEGG | Gene ID | transcript ID | log2FC | Gene name | Description | associated loci |
| --- | --- | --- | --- | --- | --- | --- |
| Phenylpropanoid biosynthesis | 9266669 | LOC_Os03g12270 | 2.926237062 | ATCAD7, CAD7, CHR, | NADPH-dependent cinnamaldehyde and hexenal reductase involved in the production of green leaf volitile compounds. | AT4G37980 |
|  | 4340333 | LOC_Os06g08580 | 3.360315189 | HCT; HYDROXYCINNAMOYL-COA SHIKIMATE/QUINATE HYDROXYCINNAMOYL TRANSFERASE | At5g48930 has been shown to encode for the hydroxycinnamoyl-Coenzyme A shikimate/quinate hydroxycinnamoyltransferase (HCT) both synthesizing and catabolizing the hydroxycinnamoylesters (coumaroyl/caffeoyl shikimate and quinate) involved in the phenylpropanoid pathway. Influence on the accumulation of flavonoids which in turn inhibit auxin transport and reduce plant growth. The mRNA is cell-to-cell mobile. | AT5G05260 |
|  | 4345717 | LOC_Os08g34790 | -1.803106142 | 4-COUMARATE: COA LIGASE 2, 4CL2, AT4CL2 | encodes an isoform of 4-coumarate: CoA ligase (4CL), which is involved in the last step of the general phenylpropanoid pathway. The catalytic efficiency was in the following (descending) order: p-coumaric acid, caffeic acid, ferulic acid, 5-OH-ferulic acid and cinnamic acid. At4CL2 was unable to use sinapic acid as substrate. | AT3G21240 |
|  | 4328552 | LOC_Os02g09490 | -1.740765861 | ATCAD5; CAD-5; CAD5; CINNAMYL ALCOHOL DEHYDROGENASE 5 | Encodes a catalytically active cinnamyl alcohol dehydrogenase which uses p-coumaryl aldehyde as a preferred substrate. It can also use sinapyl, caffeyl, coniferyl and d-hydroxyconiferyl aldehydes as substrates. | AT2G30490 |
| Starch and sucrose metabolism | 4341870 | LOC_Os06g46940 | -1.126346025 | BETA GLUCOSIDASE 41, BGLU41 | beta glucosidase 41;(source: Araport11) | AT5G54570 |
|  | 4347547 | LOC_Os09g33710 | 5.317130325 | BETA GLUCOSIDASE 11, BGLU11 | beta glucosidase 11;(source: Araport11) | AT1G02850 |
|  | 4338556 | LOC_Os05g30250 | -1.299243498 | BETA GLUCOSIDASE 11, BGLU11 | beta glucosidase 11;(source: Araport11) | AT1G02850 |
|  | 4340890 | LOC_Os06g21570 | 2.792246449 | BETA GLUCOSIDASE 17, BGLU17 | beta glucosidase 17;(source: Araport11) | AT2G44480 |
|  | 4336391 | LOC_Os04g43410 | 1.249647522 | BETA-GLUCOSIDASE 47, BGLU47 | beta-glucosidase 47;(source: Araport11) | AT4G21760 |
|  | 4345996 | LOC_Os08g39870 | 1.496434602 | BETA GLUCOSIDASE 17; BGLU17 | beta glucosidase 17;(source: Araport11) | AT1G02850 |
|  | 4336145 | LOC_Os04g39880 | -1.142104739 | BETA GLUCOSIDASE 12; BGLU12 | beta glucosidase 12;(source: Araport11) | AT2G44450 |
| Protein processing in endoplasmic reticulum | 4347405 | LOC_Os09g30418  LOC_Os09g30438 | -1.613084635 | ATHSP90.2; EARLY-RESPONSIVE TO DEHYDRATION 8; ERD8; HEAT SHOCK PROTEIN 90.2; HEAT SHOCK PROTEIN 81-2; HEAT SHOCK PROTEIN 81.2; HEAT SHOCK PROTEIN 90.2; HSP81-2; HSP81.2; HSP90.2 | A member of heat shock protein 90 (HSP90) gene family. Expressed in all tissues and abundant in root apical meristem, pollen and tapetum. Expression is NOT heat-induced but induced by IAA and NaCl. Interacts with HsfA1d in the cytosol and the nucleus and negatively regulates HsfA1d. Did not bind to AtHsfA4c. The mRNA is cell-to-cell mobile. | AT4G24190 |
| Organismal Systems | 4351825 | LOC_Os12g12730 | 5.424726188 | CALMODULIN LIKE 7; CML7; RHS1; ROOT HAIR SPECIFIC 1 | EF hand calcium-binding protein family;(source: Araport11) | AT1G05990 |
| Ascorbate and aldarate metabolism | 4348636 | LOC_Os10g28200 | 1.005001395 | GDP-D-MANNOSE 3; GME | Encodes a protein with GDP-D-mannose 3',5'-epimerase activity. The enzyme is involved in ascorbate biosynthesis. It catalyzes the conversion of GDP-D-mannose to GDP-L-galactose. | AT2G39770 |
| Metabolism | 4329643 | LOC_Os02g34810 | 1.00478158 | TAPX; THYLAKOIDAL ASCORBATE PEROXIDASE | EEncodes a chloroplastic thylakoid ascorbate peroxidase tAPX. Ascorbate peroxidases are enzymes that scavenge hydrogen peroxide in plant cells. Eight types of APX have been described for Arabidopsis: three cytosolic (APX1, APX2, APX6), two chloroplastic types (stromal sAPX, thylakoid tAPX), and three microsomal (APX3, APX4, APX5) isoforms. | AT1G77490 |
| Ascorbate and aldarate metabolism | 4341305 | LOC_Os06g36560 | -1.883857345 | MIOX1, MYO-INOSITOL OXYGENASE 1 | Encodes MIOX1. Belongs to myo-inositol oxygenase gene family. | AT1G14520 |
